# Biochar Application Alleviated Negative Plant-Soil Feedback by Modifying Soil Microbiome

**DOI:** 10.3389/fmicb.2020.00799

**Published:** 2020-04-29

**Authors:** Wenpeng Wang, Zhuhua Wang, Kuan Yang, Pei Wang, Huiling Wang, Liwei Guo, Shusheng Zhu, Youyong Zhu, Xiahong He

**Affiliations:** ^1^State Key Laboratory for Conservation and Utilization of Bio-Resources in Yunnan, Yunnan Agricultural University, Kunming, China; ^2^School of Landscape and Horticulture, Southwest Forestry University, Kunming, China

**Keywords:** biochar amendment, microbial community, soil-borne disease, negative plant-soil feedback, *Panax notoginseng*

## Abstract

Negative plant-soil feedback (NPSF) frequently cause replant failure in agricultural ecosystems, which has been restricting the sustainable development of agriculture. Biochar application has appealing effects on soil improvement and potential capacity to affect NPSF, but the process is poorly understood. Here, our study demonstrated that biochar amendment can effectively alleviate the NPSF and this biochar effect is strongly linked to soil microorganism in a sanqi (*Panax notoginseng*) production system. High-throughput sequencing showed that the bacterial and fungal communities were altered with biochar amendment, and bacterial community is more sensitive to biochar amendment than the fungal community. Biochar amendment significantly increased the soil bacterial diversity, but the fungal diversity was not significantly different between biochar-amended and non-amended soils. Moreover, we found that biochar amendment significantly increased the soil pH, electrical conductivity, organic matter, available phosphorus, available potassium, and *C*/*N* ratio. The correlation analysis showed that these increased soil chemical variables have a significantly positive correlation with the bacterial diversity. Further analysis of the soil microbial composition demonstrated that biochar soil amendment enriched the beneficial bacterium *Bacillus* and *Lysobacter* but suppressed pathogens *Fusarium* and *Ilyonectria*. In addition, we verified that biochar had no direct effect on the pathogen *Fusarium solani*, but can directly enrich biocontrol bacterium *Bacillus subtilis*. In short, biochar application can mitigate NPSF is mostly due to the fact that biochar soil amendment modified the soil microbiome, especially inhibited pathogens by enriching beneficial bacterium with antagonistic activity against pathogen.

## Introduction

Feeding a growing population and preventing the change of climate have become two great challenges facing humanity (Smith et al., 2013). The development of environmentally friendly and sustainable agricultural practices should be seriously considered to reduce the impact of land utilization on ecosystem services (Chaparro et al., 2012; Kolton et al., 2016). Faced with the limited land resources, particularly arable land, sustainable soil management has become a prerequisite for the future agriculture (Toju et al., 2018). Healthy soil usually has the ability to sustain plant health and promote plant productivity (Doran, 2002). Maintaining and promoting soil health and quality is an important guarantee and prerequisite for the development of sustainable agriculture. However, in agricultural production, same crop is often repeatedly planted in the same soil, which resulting in severe deterioration of soil and the problem of soil sickness (Huang et al., 2013; Liu et al., 2019). Soil sickness often arises with the increase of soil-borne diseases and reduction in crop yield when the one crop was cultivated on the same soil successively and that is a typical negative plant-soil feedback (NPSF) (Huang et al., 2013). NPSF is a phenomenon in which soil quality changes with plants growth, which in turn is detrimental to the growth of plants in the same soil (Bever, 1994; Yang et al., 2019). NPSF play an key role in soil sickness, which is the main factor limiting the sustainable use of soil in agricultural production systems (Huang et al., 2013). Alleviating the process of NPSF is the pivotal step for maintaining soil health and may be helpful for sustainable soil management and development of agriculture (Yang et al., 2019).

Biochar is a carbon-rich solid that produced by the pyrolysis of biomass in the condition of oxygen-limited and has attracted extensive attention due to its role in soil improvement (Beesley et al., 2011; Zhu et al., 2017). Recently, biochar have been widely applied to soil with the advantages in carbon storage, soil fertility and quality, contaminant fixation (Chen et al., 2008; Cao et al., 2009; Jeffery et al., 2013; Zhu et al., 2017) and have shown the potential capability to suppress plant diseases (Elad et al., 2010; Elmer and Pignatello, 2011; Jaiswal et al., 2014a, b, 2017) and enhance plant productivity (Spokas et al., 2012; Biederman and Harpole, 2013; Liu et al., 2013; Kolton et al., 2016). These effects of biochar may be mostly dictated by modifying soil microbial community structures and activity with the changes of microbial habitats and available nutrients (Kolton et al., 2016; Zhu et al., 2017). Meeting the twin challenges of growing food demand and climate change, it is imperative to bring environmental practices for sustainable farming. Interestingly, the previous studies have reported that biochar amendment also has potential role for mitigating climate change by long-term sequestration of carbon and influencing greenhouse gas fluxes in soil, and biochar application has been recommended as an effective countermeasure to reduce emissions of soil nitrous oxide and methane (Kolton et al., 2016; Wu et al., 2019). Moreover, there is a concerted understanding of nutrient imbalance, the accumulation of autotoxins (including ginsenosides and phenolic acids) and the changes of soil microbes, especially the enrichment of soil-borne pathogens are considered as the major driving factors of NPSF (Mangan et al., 2010; Zhou et al., 2018; Luo et al., 2019). Coincidentally, a previous study has reported that biochar application is an efficient practice for improving soil physical, chemical and biological properties (Saifullah et al., 2018). In the presence of biochar, the beneficial changes of soil property were often observed. The addition of biochar increased supplementation and reduced leaching of nutrients (Kończak and Oleszczuk, 2018; Yang et al., 2019) and biochar amended soils had lower soil bulk density, larger specific surface areas, greater water retention, higher cation exchange capacities and pH values relative to the un-amended soils (Laird et al., 2010). Biochar also showed a high adsorption capacity for autotoxic ginsenosides and a variety of organic and inorganic contaminants, and significantly reduced the toxicity of contaminants and autotoxins present in soils (Beesley et al., 2011; Kończak and Oleszczuk, 2018; Yang et al., 2019). There is an ubiquitous phenomenon that biochar amendment can alter soil microbial community compositions, increase microbial diversity and metabolic activity in the soil (Kolton et al., 2016; Li et al., 2019). Furthermore, biochar reduced the colonization and survival of pathogens in soil, but increased some plant growth promoting and biocontrol microorganisms (Jaiswal et al., 2017). According to the previously described potential benefits of biochar as a soil amendment, it might be hypothesized that biochar application may be considered as an environmental practice to mitigate the process of NPSF for sustainable farming in agricultural ecosystem.

As a member of the Araliaceae family and important herbal medicines, sanqi [*Panax notoginseng* (Burk.) F. H. Chen] is a typical plant which cultivation is often hampered by NPSF, expecially in continuous cropping system (Yang et al., 2015, 2019; Wei et al., 2018; Luo et al., 2019). Replant failure caused by NPFS in cultivated fields has been the main factor restricting the sustainable development of the sanqi production (Wei et al., 2018). The changes of soil microbial composition and diversity, especially the accumulation of soil-borne pathogens are regarded as the main driving factor of NPSF in continuous sanqi cropping system (Yang et al., 2015; Dong et al., 2016; Wei et al., 2018; Luo et al., 2019). Here, sanqi was used as the model plant to clarify the effect of soil biochar amendment on NPSF. We first evaluated the effect of biochar amendment on sanqi seedling survival, soilborne disease, culturable microorganisms, and soil properties to check the effectiveness of biochar application to mitigate NPSF in an continuous sanqi cropping system. Then we studied the correlation between the biochar effect and soil microorganism, and analyzed the soil microbial community in response to biochar amendment by high-throughput amplicon sequencing. Moreover, we explored and validated further the relationships between the soil-borne pathogen survival in soil and biochar amendment. Understanding the process of biochar application act on NPSF may help us better manage soil for sustainable agriculture through biochar soil amendment to reduce chemical fungicide application. Based on these studies, we expected to decipher the effect and underlying mechanism of biochar amendment on NPSF and to provide useful information for sustainable soil management and agricultural ecological systems.

## Materials and Methods

### Experimental Design and Conditions

In order to evaluate the effect of biochar soil amendment on the NPSF as well as the soil microbiome and seedling growth, a pot experiment was carried out in a greenhouse located at the experimental station of Yunnan Agricultural University, Xundian County, Yunnan, China (103.13°E, 25.67°N; altitude of 1,880 m). The planting soil was collected from native wasteland without cultivation, and was passed through 2 mm mesh to pick out the observable vegetations and stones for further experiments. The soil chemical characteristics were pH: 6.1; electrical conductivity (EC): 76.6 μS cm^–1^; organic matter (OM): 27.47 g kg^–1^; total nitrogen (TN): 1.15 g kg^–1^; alkali-hydrolysable nitrogen (AN): 112.98 mg kg^–1^; available phosphorus (AP): 4.78 mg kg^–1^; available potassium (AK): 43.79 mg kg^–1^. The biochar, made from wheat straw at 500°C pyrolysis temperature (Yunan Windsail Agricultural Tech CO., Ltd.), was used on the present research. The most pertinent characteristics of the biochar were pH: 9.47, 7463 μs cm^–1^ EC, 233.40 mg kg^–1^ OM, 1.59 g kg^–1^ TN, 95.55 mg kg^–1^ AN, 349.34 mg kg^–1^ AP and 5458.32 mg kg^–1^ AK (Supplementary Table S6). Healthy sanqi seedlings from the nursery base at the experimental station were carefully collected from the soil and transplanted to each plastic container pot (10 cm × 10 cm × 10 cm) containing the planting soil that was previously homogenized with or without biochar (0, 0.5, and 2%; w/w) at the beginning of June. Each treatment included ten replicates (pots) with five sanqi seedlings per replicate. All pots were placed in the greenhouse that allowed 10% light transmission and maintained temperatures below 30°C for sanqi growth, and was conducted as a randomized complete block design (Wei et al., 2018; Yang et al., 2019). The plants were irrigated three times a week and were the same in all treatments. The experiment was repeated the following year, and no fertilizer was used in all experiments.

### Evaluating the Effects of Biochar Amendment on Sanqi Growth and Disease

In order to examine the effects of biochar on the sanqi growth, the sanqi seedling survival rate was evaluated for each replicate in December. The roots of sanqi were harvested and washed with water, and disease severity was assessed by examining the roots as previously described (Wei et al., 2018). Briefly, 0: no disease; 1: necrotic lesions less than 10% of taproot; 2: approximately 10–30% of taproot cankered; 3: approximately 30–50% of taproot cankered; 4: approximately 50–75% of taproot blackened; 5: more than 75% of taproot blackened, dead plant plus number of missing. Five plants were used to calculate the sanqi seedling survival rate and disease index of each replicates.

### Soil Sampling

After sanqi harvest, soil samples were collected from each treatment according to a previously described method (Wei et al., 2018). Briefly, the soil samples were randomly collected from ten pots and mixed into three biological replicates of each treatment. The soil samples were transported to the laboratory and passed through a 2 mm sieve to remove plant debris. Then all soil samples were divided into three parts, respectively: one part was placed into 5 ml centrifuge tube and stored in a −80°C freezer for DNA extraction and further analyses; the second part was sealed in a plastic zip-top bag and stored at 4°C for microbiological isolation and culture within 1 week; the third part was air-dried and sealed in a plastic zip-top bag at room temperature for soil chemical analysis.

### Soil Chemical Property Analyses

The soil samples air-dried were used for soil chemical analysis as previously described methods. The soil pH was measured using the pH meter (AS800, AS ONE CORPORATION, China) in a 1:2.5 soil/water (w/v) suspension. Electrical conductivity was determined using the conductivity meter (MP513, Shanghai San-Xin Instrumentation Inc., China) in a 1:5 soil/water (w/v) suspension. The contents of soil total nitrogen, available nitrogen, available phosphorus and available potassium were determined as previously described (Liu et al., 1996). Soil organic matter was assayed using dichromate wet combustion according to a previously described method (Falco et al., 2004). The soil samples of each treatment with three replications were tested, respectively.

### Evaluating the Effect of Biochar Amendment on Negative Sanqi-Soil Feedback in Continuous Cropping System

The effect of biochar amendment on negative sanqi-soil feedback in continuous cropping system was determined referring to previously described methods (Mendes et al., 2011; Wei et al., 2018). Briefly, the consecutive soil from each treatment was divided into two subsamples which included five replications (pots) per subsample. One was transferred to a steel tray and placed in an electrically heated drying cabinet (MDL115, Binder, Germany) at 80°C for 1 h, and the other was not treated. Then, sanqi seeds were sown in the soils with the density of 3 cm × 3 cm and there was nine sanqi seeds each pot. Each treatment included five replications and was conducted as a randomized complete block design. All treatments were placed in the same greenhouse as the year before. The number of seed germination, seedling survival and dead plants were recorded twice a month after seedling emergence. The seed germination rate, seedling survival rate and the incidence of seedling wilt were counted to evaluate the effect of biochar amendment on negative plant-soil feedback in the sanqi continuous cropping system as previously described (Wei et al., 2018; Yang et al., 2019).

### DNA Extraction, High-Throughput Sequencing

Total genome DNA from above-mentioned three of soil samples per treatment stored in a −80°C freezer was extracted using PowerSoil^®^ DNA Isolation Kit (MO BIO Laboratories, Inc., Carlsbad, CA, United States) in accordance with the manufacturer’s instructions. The V3–V4 regions of the bacterial 16S rRNA gene and ITS2 region of the fungal internal transcribed spacer were amplified, respectively, with the primer sets 341F/806R (Wei et al., 2018) and ITS3-2024F/ITS4-2409R (Orgiazzi et al., 2012), by an ABI GeneAmp^®^ 9700 PCR thermocycler (ABI, CA, United States) as follows: initial denaturation at 95°C for 3 min, followed by 27 cycles of denaturing at 95°C for 30 s, annealing at 55°C for 30 s and extension at 72°C for 45 s, and single extension at 72°C for 10 min, and end at 4°C. The PCR mixtures contain 5 × *TransStart* FastPfu buffer 4 μL, 2.5 mM dNTPs 2 μL, forward primer (5 μM) 0.8 μL, reverse primer (5 μM) 0.8 μL, *TransStart* FastPfu DNA Polymerase 0.4 μL, template DNA 10 ng, and finally ddH2O up to 20 μL. The PCR product was extracted from 2% agarose gel and purified using the AxyPrep DNA Gel Extraction Kit (Axygen Biosciences, Union City, CA, United States) according to manufacturer’s instructions and quantified using Quantus^TM^ Fluorometer (Promega, United States). Purified amplicons were sequenced by the Illumina MiSeq platform of Novogene Corporation (Beijing, China) according to the standard protocols. The generated raw reads were deposited into the Sequence Read Archive (SRA) database of National Center for Biotechnology Information (NCBI) (Accession Number: PRJNA578849). Quality-filtering on the raw reads were performed according to the Cutadapt quality controlled process (Martin, 2011). The reads were truncated at any site receiving an average quality score of <20 over a 50 bp sliding window, and the truncated reads shorter than 50 bp were discarded. Then, the UCHIME algorithm was used to detect and remove the chimera sequences according to previous descriptions (Edgar et al., 2011; Haas et al., 2011). The sequences of 16s rRNA gene with average length of 412 bp and ITS gene with average length 309 bp were finally obtained (Supplementary Tables S1, S2). The Uparse software (Uparsev7.0.1001) was used for sequences analysis according to a previous description (Edgar, 2013). The sequences were assigned to the same OTUs with ≥97% similarity and representative sequence was screened for further annotation. In order to annotate taxonomic information of representative sequence, the Silva Database (Version132) was used based on Mothur algorithm for the bacterial 16S rRNA gene (Quast et al., 2012) and the Unite Database (Version7.2) was used based on Blast algorithm for the fungal ITS2 region (Urmas et al., 2013). Then, the data of each sample were processed by normalization based on the minimum data in the sample (Luo et al., 2019). Further analyses of alpha and beta diversity were performed basing on the OTUs abundance information which were normalized.

### Effects of Biochar Amendment on Culturable Microorganisms in Soil

The effects of biochar on the numbers of culturable fungi, bacteria and actinomycetes survival in soil were evaluated on selective media by serial dilution plating according to previous descriptions (Xie et al., 2015; Yang et al., 2019). Briefly, 10 g of soil sample were added to 90 mL of sterilized water. The soil suspension was decimally diluted after homogenization for 20 min, and 100 μl of the solutions were plated on the medium of rose bengal agar for fungi, beef extract peptone for bacteria and Gauze’s medium No. 1 for actinomycetes, respectively. After incubation at 28°C for 2–7 days, counting the colony forming units (CFU) and the results were expressed as CFU per gram of dry soil.

### Isolation and Identification of the Pathogen and Antagonistic Bacteria

The colony numbers with the typical morphology of *Fusarium* spp. and *Bacillus* spp. from the selective medium (Komada, 1975) and beef extract peptone medium, respectively, were recorded and counted as CFU per gram of dry soil according to a previous description (Wei et al., 2018). The representative isolates of *Fusarium* on the RB plates were selected and a pathogenicity test was performed on sanqi roots according to previous descriptions (Mao et al., 2014; Wei et al., 2018; Luo et al., 2019). The representative isolates of *Bacillus* on the BEP plates were selected and the antagonistic activity against an isolate of *Fusarium* with pathogenicity on sanqi was tested according to previous descriptions (Mendes et al., 2011; Tu et al., 2013; Luo et al., 2019). Then, the isolates of *Fusarium* with strongest pathogenicity and *Bacillus* with highest antagonistic activity were further screened on the basis of the pathogenicity and antagonistic activity test, and identified by analyzing ITS and 16S rRNA gene sequence, respectively, as previously described (White et al., 1990; Wei et al., 2018; Luo et al., 2019). The generated sequences were submitted to the NCBI GenBank and compared with published gene sequences from the NCBI website using the BLAST algorithm. The software MEGA 7.0 was used to construct neighbor-joining (NJ) trees and generate maximum composite distance matrices according to standard parameters (Kumar et al., 2016).

### Direct Effect of Biochar on a Pathogenic *Fusarium* and an Antagonistic Bacillus

Direct effect of biochar on the isolate selected and identified as pathogen on the basis of above test was assessed by measuring radial hyphal growth *in vitro* contact assay as previously described (Jaiswal et al., 2014b, 2017). Briefly, growing media (Potato Dextrose Agar) was amended biochar with varying concentrations (0, 0.5, 1, 2, and 3%, w:v). Agar plugs (5 mm) covered with mycelium of the isolate were placed in the center of growing media and incubated at 25°C. Following the incubation period (6 days), mycelial growth of the isolate was measured as the average of two perpendicular diameters of each thallus. In order to determine the direct effect of biochar on the antagonistic bacteria, a modified method of 3-(4,5-dimethylthiazol-2-yl)-2,5-diphenyltetrazolium bromide (MTT) assay was used in our study according to previous description (Shi et al., 2007; Grela et al., 2018). Briefly, the bacterial suspension was prepared and incubated inoculated in nutrient broth with biochar amendment at varying concentrations (0, 0.5, 1, 2, and 3%, w/v) and incubated on rotary incubator shaker (150 rpm; 27 ± °C). After incubating for 24 h, 200 μL of bacterial suspensions and 1 ml of MTT solution (5 mg/ml) were mixed and incubated in the dark at 30°C for 1 h. Then, the supernatant was removed by centrifuging and 1 ml of dimethylsulfoxide (DMSO) was added to dissolve purple crystals of formazan. Finally, all samples were passed through a 0.22 μm sieve and the absorbance of filtrate was measured in a spectrophotometer at a wavelength of 510 nm. All tests were performed in triplicate.

### Effect of Biochar on Pathogen and Antagonistic Bacteria Survival in Soil

The soil previously collected from the native wasteland was divided into two subsamples. One was used directly as the natural soil (unsterilized soil) and amended biochar with varying concentrations (0, 0.5, 1, 2, and 3%, w/w). The other was treated by fully sterilizing at 121°C for 30 min as the sterilized soil and then amended biochar with varying concentrations (0, 0.5, 1, 2, and 3%, w/w). The isolates selected and identified as pathogen (*Fusarium solani*) and antagonistic bacteria (*Bacillus subtilis*) on the basis of above tests were equally inoculated in the soils containing biochar at varying concentrations to assess the effects of biochar on pathogen and antagonistic bacteria survival in soil, respectively. Assessment of pathogen and antagonistic bacteria populations were carried out using quantitative real-time polymerase chain reaction (q-PCR) after 60-day incubation at room temperature and were expressed as number of DNA copy g^–1^ soil. Total genomic DNA of soil samples was extracted by using a PowerSoil^®^ DNA Isolation Kit (MO BIO Laboratories, Inc., Carlsbad, CA, United States) in accordance with the manufacturer’s instructions. The ITS region of *F. solani* were amplified using the modified primer sets ZF (5′-ACGCCGTCCCTCAAATACAG-3′)/ZR (5′-GAAGTTGGGTGTTTTACGGCA-3′) according to previous descriptions (Li et al., 2008; Bernal-Martínez et al., 2012) and the 16S rRNA genes of *B. subtilis* were amplified using the primer sets BF (5′-CCTACGGGAGGCAGCAGTAG-3′)/BR (5′-GCGTTGCTCCGTCAGACTTT-3′). Real-time q-PCR was performed on a LightCycler^®^ 96 Real-Time PCR System (Roche, Basel, Switzerland) according to previous descriptions (Zhao et al., 2017; Wei et al., 2018). Briefly, The PCR products were purified, cloned into the pMD19-T vector (TaKaRa, Tokyo, Japan) and transformed into competent Escherichia coli TOP10 cells (Invitrogen, Carlsbad, CA, United States). The positive clones were selected and sequenced. The extracted plasmid DNA was used to build a standard curve. The q-PCR amplification was performed as follows: initial denaturation at 95°C for 10 min, followed by 45 cycles of denaturing at 95°C for 10 s, annealing at 60°C for 10 s and elongation at 72°C for10 s. Fluorescence was detected at the end of the elongation phase for each cycle. After the real-time PCR assay, the specificity of the amplification was verified by a melting curve analysis which was obtained by heating the mixture to 95°C, cooling to 65°C (15 s), and slowly heating to 95°C at 0.1 C s^–1^ with continuous measurement of fluorescence. The PCR mixtures contain LightCycler 480 SYBR Green I Master (Roche Diagnostics Gmbh, Germany) 10 μL, ddH_2_O 3 μL, forward primer (10 μM) 1 μL, reverse primer (10 μM) 1 μL, and template DNA 5 μL.

### Statistical Analysis

The significant differences in the seed germination and seedling survival rate, chemical properties of soils, disease index and the incidence of disease were calculated at the 5% level using one-way analysis of variance (ANOVA) by SPSS version 18.0 software (SPSS Inc. Chicago, IL, United States). The data of microbial taxa mainly were analyzed using R software (Version 2.15.3). The means of alpha diversity indices were compared between treatments by the Tukey’s honestly significant difference (HSD) test. For the relative abundances of the dominant microbial genus, means were compared between treatments by the Welch’s *t*-test. The soil chemical properties and the microbial alpha diversity indices were correlated by employing Pearson’s correlation coefficient. For beta diversity analysis, the principal coordinate analysis (PCoA) based on the weighted unifrac distance and the unweighted hierarchical clustering was calculated using R software to visualize the community similarity. The analysis of similarity (ANOSIM) based on Bray–Curtis distance was performed using the free online platform of Majorbio Cloud Platform.^1^

## Results

### Biochar Increased Sanqi Seed Germination, Seedling Survival and Suppressed Soil-Borne Disease in Continuous Cropping System

**FIGURE 1:**
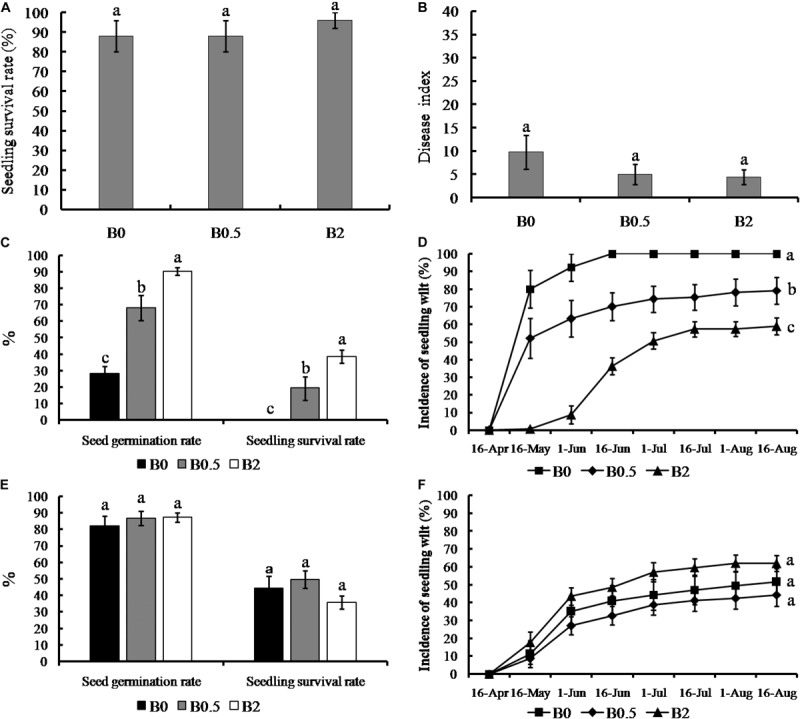
Effect of biochar amendment on seedling survival, root rot disease severity and plant-soil feedback of sanqi. Panel **(A,B)** shows the effect of biochar on seedling survival rate and the disease index of sanqi root rot in the uncultivated soil, respectively; **(C)** shows the seed germination and seedling survival rate of sanqi in consecutively cultivated soil; **(D)** shows the disease progress curves of sanqi seedling wilt in consecutively cultivated soil; **(E)** shows the seed germination and seedling survival rate in consecutively cultivated soil with heat-treated; **(F)** shows the disease progress curves of sanqi seedling wilt in consecutively cultivated soil with heat-treated. B0, B0.5, and B2 stand for biochar soil amendment at a concentration of 0, 0.5, and 2% (w/w), respectively. All data are presented as the mean ± standard errors (SE) and bars indicate SE. The different letters above the error bars indicate significant differences among the treatments (*p* < 0.05; *n* = 5; Duncan’s multiple range test).

The soil without sanqi cultivation previously showed the ability to sustain sanqi growth, which the seedling survival rates of all treatments were more than 85% and the disease indexs of root rot were less than 10 (Figures 1A,B). Moreover, the effects of biochar amendment at the concentrations of 0.5 and 2% on the sanqi growth both were not significant in the uncultivated soil, which seedling survival rates and disease index of root rot did not show significant differences between biochar-amended and non-amended (Figures 1A,B). In order to identify the effectiveness of biochar amendment on NPSF, we analyzed further sanqi seed germination rate, seedling survival rate and the incidence of seedling wilt in the sanqi seedling continuous cropping system (Figures 1C–F). The seed germination and seedling survival rate was increasing with the increasing concentrations of biochar applications (0, 0.5, and 2%) when the sanqi was cultivated consecutively (Figure 1C). The seed germination rate and seedling survival rate were significantly increased when biochar amendment at concentrations of 0.5 and 2% compared without biochar amendment (*p* < 0.05). In consecutively cultivated system, the incidence of seedling wilt was increasing with the growth of sanqi (Figure 1D). The incidences of seedling wilt in soil consecutively cultivated with biochar amendment at the concentration of 0.5 and 2% were significantly decreased compared to that without biochar amendment (*p* < 0.05). However, the seed germination rate, seedling survival rate and the incidence of seedling wilt both were not significant among all concentrations of biochar amendment (0, 0.5, and 2%) when sanqi was cultivated consecutively in the soil heat-treated (Figures 1E,F).

### Effects of Biochar Amendment on Soil Chemical Properties

The biochar amendment increased the values of soil AP, AK, pH, EC, OM and carbon-nitrogen ratio (C/N), and these chemical properties both were significantly higher in the soil with biochar amendment at concentration of 2% compared to the soil without biochar amendment (*p* < 0.05). The effects of biochar amendment at the concentrations of 0.5 and 2% on contents of soil TN and AN were not significant (Table 1).

**TABLE 1:** Effects of biochar amendment on soil chemical properties.

**Soil properties**	**Unit**	**Treatment**		
		**B0**	**B0.5**	**B2**
Total nitrogen (TN)	g/kg	1.150.01a	1.160.00a	1.170.02a
Organic matter (OM)	g/kg	27.470.37b	28.430.73b	32.080.64a
Alkali-hydrolysable nitrogen (AN)	mg/kg	112.983.74a	111.166.08a	101.794.22a
Available phosphorus (AP)	mg/kg	4.780.35b	6.380.77b	15.593.48a
Available potassium (AK)	mg/kg	43.790.35c	115.411.27b	261.081.49a
Electrical conductivity (EC)	μS/cm	76.601.37c	98.606.32b	145.400.47a
Carbon-nitrogen ratio (C/N)		13.840.18b	14.170.36b	15.930.54a
pH		6.100.01b	6.170.05ab	6.260.03a

*All data for the effects of biochar on soil chemical properties are presented as the mean ± standard errors calculated from three biological replicates. Data in the same row followed by different letters showed significant differences (p < 0.05; n = 3).*

### Effects of Biochar Amendment on Culturable Microorganisms in Soil

The biochar amendment effectively suppressed fungi survived in the soils. The number of culturable fungi did not show significant differences between biochar amendment at concentration of 0.5 and 2%, but was significantly less in the soils with biochar amendment at concentration of 0.5 and 2% compared to the soil of without biochar amendment (*p* < 0.05). However, the biochar amendment effectively increased bacteria and actinomycetes survived in the soils, and the effectiveness increased with increasing concentration of biochar in the soil. The number of bacteria and actinomycetes both were significantly higher in the soils with biochar amendment at concentration of 0.5 and 2% compared to the soil of without biochar amendment (*p* < 0.05), and also were significantly higher in the soils with biochar at concentration of 2% compared to 0.5% (Table 2).

**TABLE 2:** Effects of biochar amendment on soil microbial communities.

**Treatment**	**Fungi (×10^3^CFU/g)**	**Bacteria (×10^6^CFU/g)**	**Actinomycetes (×10^5^CFU/g)**
B0	65.14 ± 6.97a	26.47 ± 1.87c	19.67 ± 1.83c
B0.5	36.20 ± 1.89b	35.20 ± 1.25b	31.13 ± 2.23b
B2	33.93 ± 2.84b	44.08 ± 2.49a	48.21 ± 3.49a

*Data are shown as the mean with standard error. Data in the same column followed by different letters showed significant differences (p < 0.05; n = 3). CFU, colony-forming unit.*

### Microbial Diversity and Their Relationships With Soil Chemical Properties

**FIGURE 2:**
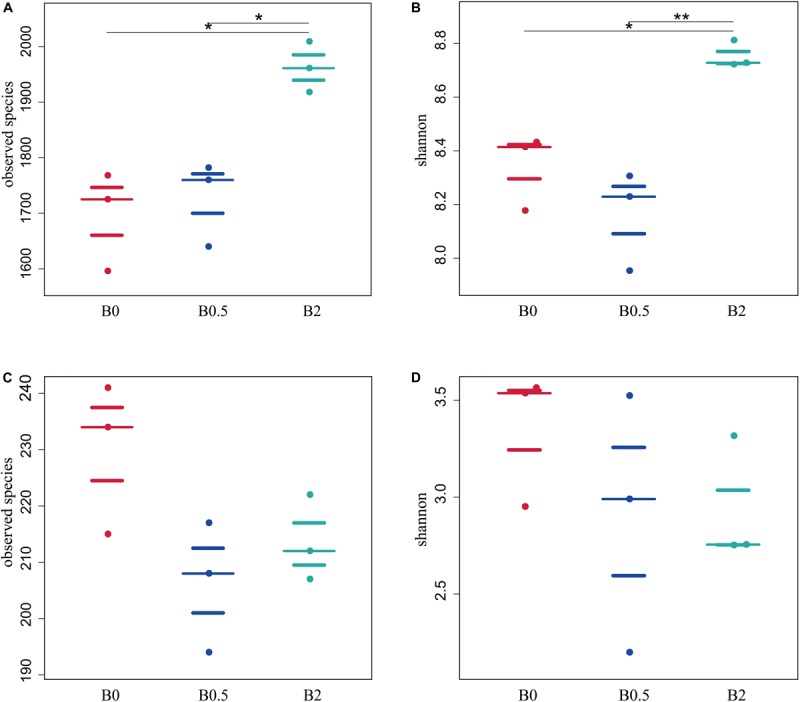
The effects of biochar soil amendment on microbial diversity index. Panel **(A,B)** shows the effects of biochar amendment on the observed species, and Shannon diversity index of bacterial community, respectively; **(C,D)** shows the effects of biochar amendment on observed species, and Shannon diversity index of fungal community, respectively. Asterisks denote significant differences between different treatments as determined by the Tukey’s honestly significant difference test (*P < 0.05, **P < 0.01; *n* = 3).

Illumina sequencing of 16S rRNA and ITS gene amplicons was performed to explore the effect of biochar soil amendment on microbial community structure and diversity. Across all soil samples, Illumina Miseq sequencing yielded 1,071,945 quality bacterial sequences and 925,965 quality fungal sequences, with 112,898–124,375 bacterial sequences (mean = 119,105) and 101,823–104,726 fungal sequences per sample (mean = 102,885) after quality-filtering, respectively (Supplementary Tables S1, S2). The sequencing depth with more than 99% coverage estimators (Supplementary Tables S2, S3) demonstrated that it was sufficient to capture the community structure and diversity of bacteria and fungi under current work. In this study, the biochar applications at concentrations of 0.5% had not significant effect on bacterial community diversity compared to those without biochar amendment. However, the bacterial diversity indexes including observed species, chao1, abundance-based coverage estimator (ACE), Shannon and Simpson were significantly higher in the soil with biochar amendment at concentration of 2% compared to those without biochar amendment (*p* < 0.05). In addition, the observed species, Shannon and Simpson index in the soil with biochar amendment at concentration of 2% was significantly higher than those with biochar amendment at concentration of 0.5% (Figures 2A,B and Supplementary Table S3). As for fungi, there were slightly lower richness and diversity in the soil with biochar addition than that without biochar amendment, while the indices of observed species, chao1, ACE, Shannon and Simpson in the soil with biochar addition did not have significant differences compared with those without biochar amendment (Figures 2C,D and Supplementary Table S4).

**FIGURE 3:**
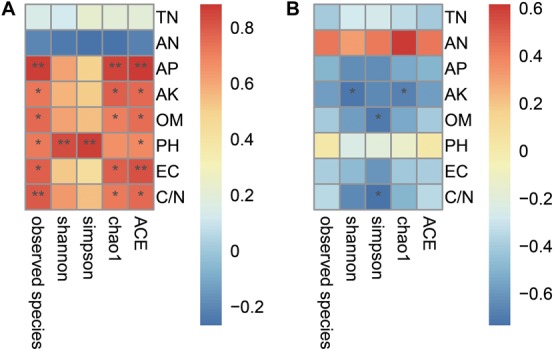
The relationships of microbial alpha diversity and soil chemical properties. Panel **(A)** showed the relationship of bacterial diversity and soil chemical properties; **(B)** showed the fungal diversity and soil chemical properties. The numbers on the right stands for the relationship between the diversity indexes and soil chemical properties based on the Pearson’s correlation analyses. Asterisks denote significant relationship between the microbial diversity indexes with the soil chemical properties (*P < 0.05, **P < 0.01).

The biochar amendment changes microbial diversity and soil environmental characteristics. In this study, Pearson’s correlation analysis revealed that the microbial diversity was affected by primary environmental characteristics (including TN, AN, AP, AK, OM, pH, EC, and C/N). As shown in Figure 3, most of soil chemical variables had a positive correlation with the bacterial diversity, but had a negative correlation with fungal diversity. The indexes of bacterial ACE and observed species were positively related to AP, AK, OM, pH, EC, and C/N (*p* < 0.05); chao1 was positively related to AP, AK, OM, EC, and C/N (*p* < 0.05); Shannon and Simpson were positively related to pH (*p* < 0.05). However, all diversity indexes of bacteria (including ACE, observed species, chao1, Shannon and Simpson) were not significantly related to TN and AN (Figure 3A). As for fungi (Figure 3B), chao1 and Shannon were negatively related to AK (*p* < 0.05), and Simpson was negatively related to OM and C/N (*p* < 0.05). In addition, all diversity indexes (including ACE, observed species, chao1, Shannon and Simpson) of fungi were not significantly related to TN, AN, AP, pH, and EC.

### Effect of Biochar Amendments on Bacterial and Fungal Community Structure

**FIGURE 4:**
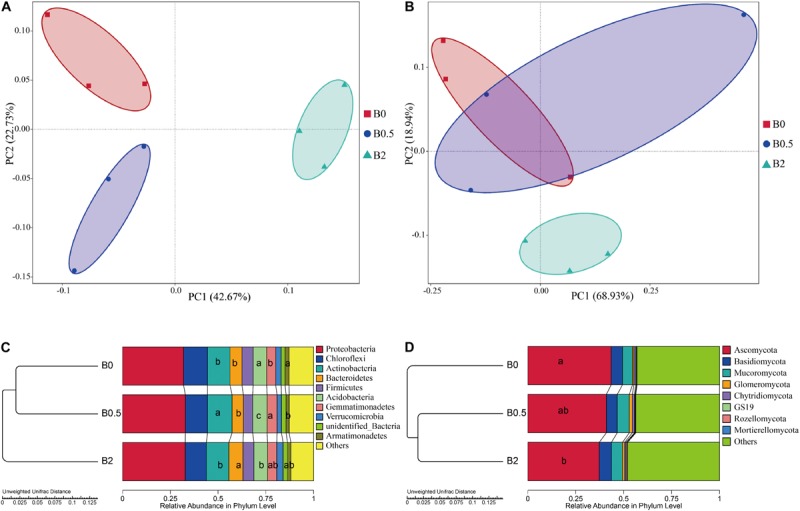
Principal coordinate analysis (PCoA), hierarchical clustering and the relative abundances of bacteria and fungi. Panel **(A,B)** are the PCoAs of the bacterial and fungal communities at operational taxonomic unit level, respectively; **(C,D)** are the unweighted hierarchical clustering and relative abundances of bacteria and fungi at phylum levels, respectively. Different letters in columns show significant differences as determined by the Duncan’s multiple range test (*P* < 0.05; *n* = 3).

The over all structural change of the bacteria and fungi was analyzed using principal coordinate analysis (PCoA) and the analysis of similarity (ANOSIM) at operational taxonomic unit (OTU) level. The bacterial community structures among the soils with biochar at the concentrations of 0, 0.5, and 2% (w/w) showed significant differences (Supplementary Table S5; ANOSIM, *r* = 0.93, *p* = 0.001). Principal coordinates analysis showed that the bacterial community structures were separated from the treatment with biochar amendment at the concentration of 2% to the treatments without biochar amendment and with biochar amendment at the concentration of 0.5% in the first axis, and from the treatment without biochar amendment to the treatment with biochar amendment at a concentration of 0.5% in the second axis (Figure 4A). The fungal community structures among the soils with different dose of biochar also showed significant differences, but were less significantly compared to the differences of the bacterial community structures (Supplementary Table S5; ANOSIM, *r* = 0.33, *p* = 0.017). Principal coordinates analysis showed that the fungal community structures were separated from the treatment with biochar amendment at the concentration of 2% to the treatments without biochar amendment and with biochar amendment at the concentration of 0.5% in the second axis, but there was not a clear segregation of the fungal community structures between the treatment without biochar amendment and the treatment with biochar amendment at the concentration of 0.5% (Figure 4B).

**FIGURE 5:**
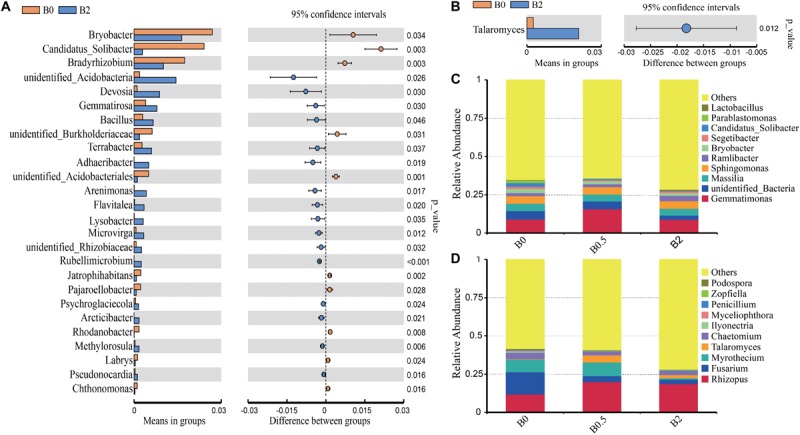
The relative abundances of bacteria and fungi at the genus level. Panels **(A,B)** showed the dominant (relative abundances > 0.1%) bacterial and fungal genera with significant differences of the relative abundances between treatments without biochar amendment and with biochar amendment at the concentration of 2% based on Welch’s *t-*test (*P* < 0.05; *n* = 3); **(C,D)** shows the relative abundances of top 10 main bacterial and fungal genera, respectively.

Biochar amendment altered the composition of bacterial and fungal communities from the phylum to genus levels. For bacterial taxa, the sequences were predominantly associated with the phyla *Proteobacteria*, *Chloroflexi*, *Actinobacteria*, *Bacteroidetes*, *Firmicutes*, *Acidobacteria*, and *Gemmatimonadetes*, and these seven phyla accounted for more than 85% of the bacterial sequences (Figure 4C). And the relative abundances of *Actinobacteria* and *Gemmatimonadetes* were significantly higher at the treatment with biochar amendment at the concentration of 0.5% than the treatment without biochar amendment (*P* < 0.05). The relative abundances of *Bacteroidetes* was significantly higher at the treatment with biochar amendment at the concentration of 2% than at the treatments without biochar amendment and with biochar amendment at the concentration of 0.5% (*P* < 0.05). The relative abundances of *Acidobacteria* was significantly higher at the treatment without biochar amendment than at the treatments with biochar amendment at the concentration of 0.5 and 2% (*P* < 0.05), and the relative abundances of *Armatimonadetes* was significantly higher at the treatment without biochar amendment than with biochar amendment at the concentration of 0.5% (*P* < 0.05). At the genus level, the relative abundances of most genera were gradually changed with biochar amendment (Figure 5C). Among those dominant bacterial genera (relative abundances > 0.1%), compared with the soil without biochar amendment, the soil with biochar amendment at the concentration of 2% had significantly higher relative abundances of 16 genera including the potential biocontrol bacterium *Bacillus* and *Lysobacter*, but had significantly lower relative abundances of 10 other genera (*P* < 0.05) (Figure 5A). For the fungal taxa, *Ascomycota*, *Basidiomycota* and *Mucoromycota* were the dominant fungal phyla in all soils, and these three phyla accounted for more than 30% of the fungal sequences (Figure 4D). Moreover, the relative abundance of *Ascomycota* was gradually reduced with the increase in biochar rates and was significantly lower in the soil with biochar amendment at the concentration of 2% than the soil without biochar amendment (*P* < 0.05) (Figure 4D). Among those dominant fungal genus (relative abundances > 0.1%), the soil with biochar amendment at the concentration of 2% showed only the relative abundance of *Talaromyces* significantly higher compared with the soil without biochar amendment (Figure 5B). Though not significant, the potential pathogens including *Fusarium* and *Ilyonectria*, showed a gradual decrease in relative abundance caused by the increased biochar rates (Figure 5D).

### Isolation and Identification of the Pathogen and Antagonistic Bacteria

**FIGURE 6:**
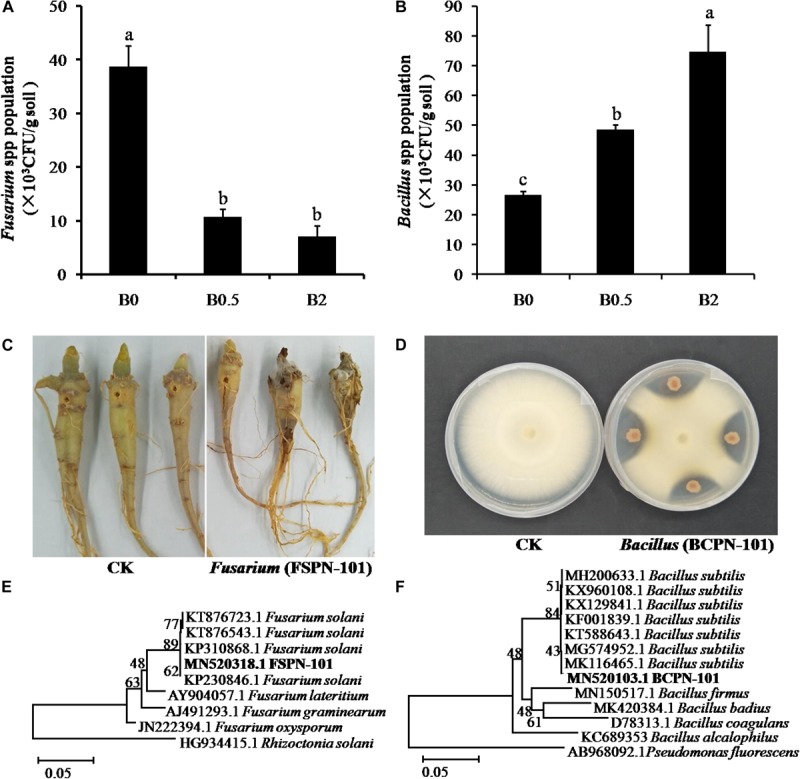
The isolation and identification of pathogen *Fusarium* and antagonistic bacteria *Bacillus.* Panel **(A)** shows the number of potential pathogen *Fusarium* isolated from different soil; **(B)** shows the number of potential beneficial bacteria *Bacillus* isolated from different soil; **(C)** shows the symptoms of sanqi root rot after inoculating *Fusarium* strain FSPN-101; **(D)** shows the antagonistic activity against *Fusarium solani* of *Bacillus* strain BCPN-10; **(E)** shows hierarchical clustering of ITS genes of the *F. solani* strain FSPN-101; **(F)** shows hierarchical clustering of 16S rDNA genes of the *Bacillus subtilis* strain BCPN-10. Different letters indicate significant differences among the treatments by Duncan’s multiple range tests (*p* < 0.05; *n* = 4).

The *Fusarium* spp. was more frequently isolated from the soil without biochar amendment than the soils with biochar amendment, and the least number was obtained from the soil with biochar at concentration of 2% (Figure 6A). The strain FSPN-101 with strong pathogenicity on sanqi root (Figure 6C) was identified as *F. solani* by ITS sequence analyses (Figure 6E) and submitted to the NCBI GenBank (Accession Number: MN520318). The amount of the isolated *Bacillus* spp. were significantly more in the soils with biochar amendment at the concentration of 0.5 and 2% than the soil without biochar (Figure 6B). The selected strain BCPN-101 showed high antagonistic activity against the isolate of *F. solani* (Figure 6D) and was identified as *B. subtilis* by 16S rRNA gene sequence analyses (Figure 6F) and submitted to the NCBI GenBank (Accession Number: MN520103).

### *In vitro* Direct Effect of Biochar Pathogen and Antagonistic Bacteria

**FIGURE 7:**
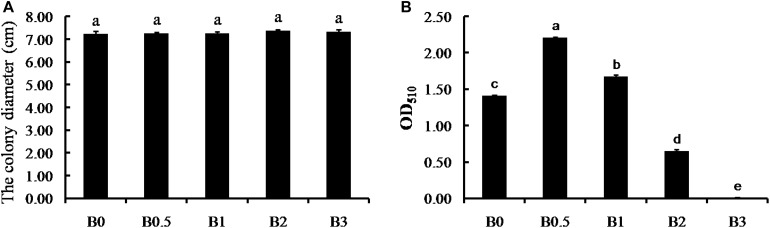
*In vitro* direct effect of biochar on pathogen and antagonistic bacteria. Panels **(A,B)** shows the direct effect of biochar on *F. solani* strain FSPN-101 and *B. subtilis* strain BCPN-101, respectively. B0, B0.5, B1, B2, and B3 represents biochar amendment at a concentration of 0, 0.5, 1, 2, and 3% (w/v). Error bars indicate the standard error, and the different letters above the bars showed significant differences (*p* < 0.05; *n* = 5).

The thallus diameters of *F. solani* strain FSPN-101 were not significantly different with biochar amendment at increasing concentrations (0, 0.5, 1, 2, and 3%), and the distinct suppression of biochar on mycelium growth was not detected *in vitro* assays (Figure 7A). For *B. subtilis* strain BCPN-101, an n-shaped response curve as a function of biochar concentration was observed (Figure 7B). It is noteworthy that the treatments with biochar amended at the concentrations of 0.5 and 1% significantly promote the *B. subtilis* growth, but the 2 and 3% concentrations significantly suppress the *B. subtilis* growth compared with the non-amended control (*P* < 0.05). The most significant promote was at 0.5% biochar amended, with the OD_510_ was significantly higher at 0.5% than at 0, 1, 2, and 3% (Figure 7B).

### Effect of Biochar on Pathogen and Antagonistic Bacteria Survival in Soil

**FIGURE 8:**
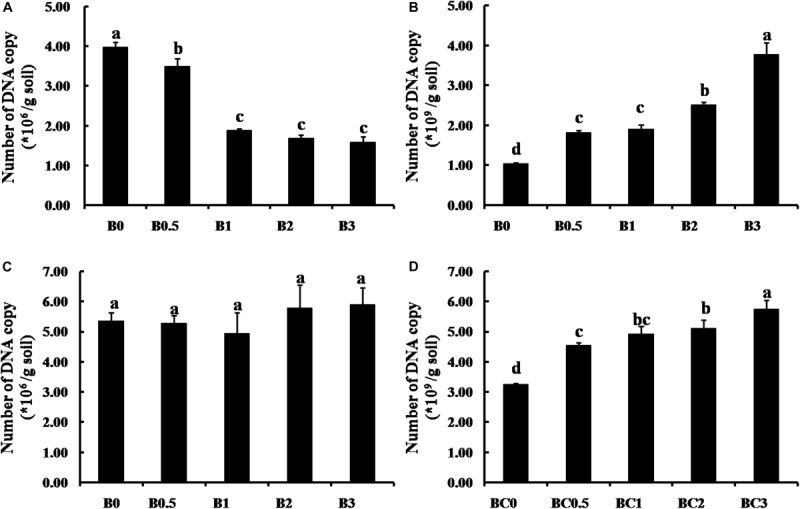
Effect of biochar on pathogen and antagonistic bacteria survival in soil. Panels **(A,B)** shows the effect of biochar on *F. solani* and *B. subtilis* in the natural soil, respectively; **(C,D)** shows the effect of biochar on *F. solani* and *B. subtilis* in the sterilized soil, respectively. B0, B0.5, B1, B2, and B3 represents biochar amendment at a concentration of 0, 0.5, 1, 2, and 3% (w/w), respectively. Error bars indicate the standard error, and the different letters above the bars showed significant differences (*p* < 0.05; *n* = 3).

The effects of biochar at increasing concentrations (0, 0.5, 1, 2, and 3%) on pathogen and antagonistic bacteria survival in soil are presented in Figure 8. In the natural soil, the number of *F. solani* decreased with increasing biochar concentrations, and *F. solani* survival in all treatments with biochar amended were significantly lower compared with the non-amended control (*P* < 0.05) (Figure 8A). However, the number of *F. solani* were not significant among all treatments, and the biochar had no the distinct suppression on *F. solani* survival in the sterilized soil (Figure 8C). For antagonistic bacteria, the number of *B. subtilis* both increased over increasing biochar concentrations in the natural soil and the sterilized soil, and the numbers of *B. subtilis* were significantly higher survival in soils with biochar amended compared with non-amended (*P* < 0.05)(Figures 8B,D).

## Discussion

Replant failure caused by NPFS in cultivated fields is a critical factor restricting the sustainable agricultural production (Huang et al., 2013; Wei et al., 2018). Biochar as an amendment to improve soil quality, revitalize degraded soil, and increase agronomic productivity have been emphasized. However, besides agronomic benefits, negative agronomic effects also have been reported in past research (Spokas et al., 2012). The suitability of biochar soil amendment depends on the biochar characteristics, soil properties, and target plants (Prapagdee and Tawinteung, 2017). In this study, the wheat straw biochar produced at 500°C as a soil amendment was evaluated for mitigating NPFS and sustainable soil management in a sanqi continuous cropping system. The result demonstrated that biochar application markedly increased the soil microbial diversity and modified microbial community structures, particularly, enriched some beneficial bacterium with antagonistic activity against pathogen. Subsequently, pathogens were significantly suppressed in the soil, and these eventually mitigated the process of negative plant-soil feedback.

### Biochar Application Can Mitigate NPSF Is Strongly Linked to Soil Microorganism

There is general consensus that the process of NPSF was complex, resulted from the interaction of plants, soil, and microorganisms, and the changes of abiotic and biotic soil properties are the major causes (Brinkman et al., 2010; Zhang et al., 2019). Previous study has reported that the soil microbial structure and the balance of nutrients were changed, and autotoxic ginsenosides were accumulated after planting sanqi. These changes limited the sanqi growth and increased diseases, eventually led to replant failure (Yang et al., 2019; Zhang et al., 2019). Biochar as a soil amendment with some nutrients remaining from carbonization process can increase the soil fertility (Schulz and Glaser, 2012; Ding et al., 2016). Previous literatures have reported the positive effect of biochar amendment on crops by directly providing nutrients or indirectly improving soil properties (Atkinson et al., 2010; Yang et al., 2019). Our results (Table 1) agreed with previous reports that biochar amendment could alter the soil chemical properties and improve the quality and fertility of soil (Laird et al., 2010; Prapagdee and Tawinteung, 2017). Moreover, some studies have reported that the populations of bacteria and actinomycetes were significantly suppressed, whereas the population of fungi showed increasing trends in the soils with sanqi growth (Dong et al., 2016; Zhang et al., 2019). The increase of bacteria and actinomycetes, and decrease of fungi can reflect the improvement of soil microbial structure (Zhang et al., 2019). In our study, the results (Table 2) are similar to previous report that biochar amendment increased the counts of culturable bacteria and actinomycetes (Jaiswal et al., 2017). These biotic and abiotic indicators were restored in biochar-amended relative to non-amended soils. In addition, previous study suggested that biochar may help ward off deleterious effects of allelochemicals (cinnamic, coumaric, and ferulic) on colonization of mycorrhizal associations and be useful in overcoming the deleterious effects of allelopathic residues in replant soils (Elmer and Pignatello, 2011). Thus, it would be suggested that biochar amendment have the potential to alleviate the NPSF for facilitating the sustainable utilization of soil in agricultural production system. Previous study also reported that biochar had a positive effect on colonization of mycorrhizal associations and this effect is independent of whether allelochemicals were added (Elmer and Pignatello, 2011). Here, we found that biochar amendment significantly increased the seed germination rate and seedling survival rate, and decreased the incidence of seedling wilt when sanqi was cultivated consecutively (Figures 1C,D). These results further confirmed the existence of the biochar effect, which biochar amendment can effectively alleviates the NPSF in sanqi production system. However, this biochar effect was not observed when sanqi was consecutively cultivated in the soil heat-treated at 80°C for 1 h (Figures 1E,F). Although sterilization may cause partial change of soil properties, appropriate high temperature treatment of soil is still exploited by many researchers to suggest that biological agents were key factors for NPSF (Mangan et al., 2010; Wei et al., 2018; Luo et al., 2019). In previous study, the suppressive soil heat-treated at 80°C loss of suppressiveness also indicated that disease suppressiveness toward pathogen was microbiological in nature (Mendes et al., 2011). Hence, the loss of the biochar effect resulted from the heat treated at 80°C can be ascribed to the absence of soil microbes. This result indicates that biochar application can mitigate the process of NPSF is strongly linked to soil microorganism in the sanqi planting system. It is similar to previous study that the biochar effect can be dictated by the changes of microbiome (Kolton et al., 2016).

### Biochar Amendment Altered the Soil Microbial Community

In this study, biochar amendment significantly increased the bacterial community richness and diversity, similar to earlier observations (Kolton et al., 2016; Jaiswal et al., 2017). This increased diversity may be mostly ascribed to the diverse organic compounds and the porous structure stem from the biochar, which provide nutrition and expand ecological niches for diverse microbes (Saito and Marumoto, 2002; Warnock et al., 2007; Kolton et al., 2016). Previous reports have suggested the enhancements of soil microbial richness and diversity were beneficial to ecosystem functioning, which can recover more quickly under stress conditions (Yin et al., 2000; Kolton et al., 2016). High soil microbial richness and diversity have been associated with plant resilience and protection against soil-borne pathogen invasion (Jousset et al., 2011; Mendes et al., 2011; Jaiswal et al., 2014a). Here, we suggested that the positive effect of biochar amendment on soil-borne diseases repression may associate with the higher bacterial richness and diversity. In addition, previous studies have demonstrated that the soil microbial community structures and diversity could be affected by changes of soil properties (Rousk et al., 2010; Zhang et al., 2017; Wei et al., 2018). The pearson’s correlation analysis revealed that the most of soil chemical variables had a significantly positive correlation with the bacterial diversity, but showed a slightly negative correlation with fungal diversity (Figure 3). This data implies that the changes of soil chemical properties induced by biochar amendment play more important roles in modifying the bacterial community diversity than fungal community diversity. The analysis of the relative abundances of microbe, ANOSIM and PCoA all confirmed the communities of bacterial and fungal both were distinctly modified by biochar amendment, and showed bacterial community structures are more sensitive to biochar soil amendment than fungal community. *Ilyonectria* spp. and *Fusarium* spp. have been well known as the main the soil-borne pathogens caused root rot of sanqi (Mao et al., 2014; Wei et al., 2018). *Bacillus* (Ongena and Jacques, 2008; Tahir et al., 2017) and *Lysobacter* (Islam et al., 2005; Islam, 2010) have been well known as the effective biocontrol bacteria, and widely used to protect against soil-borne disease. Some studies have demonstrated that after sanqi was planted, the NPSF was caused by the suppression of antagonist microbes with potential biocontrol ability and the accumulation of soil pathogens, and lead to the replanting problem (Miao et al., 2016; Dong et al., 2018; Luo et al., 2019). In our study, biochar amendment significantly increased the relative abundances of potential biocontrol bacterium *Bacillus* and *Lysobacter* (Figure 5A). In addition, the relative abundance of potential pathogens including *Fusarium* spp. and *Ilyonectria* spp. gradually decreased with the increased biochar rates (Figure 5D). Here, it is suggested that the alleviated NPSF with higher seedling survival rates and the lower disease incidence in the treatments with biochar amendment can be due to the suppression of pathogen.

### Biochar Amendment Inhibited Pathogen by Enriching Beneficial Microbes

Previous reports have demonstrated that the effects of biochar on microbe are diverse with several different mechanisms (Zhu et al., 2017). In this study, biochar amendment significantly increased the amount of isolated potential biocontrol bacteria *Bacillus* spp. in the soil. In addition, biochar amendment significantly promoted the growth of *B. subtilis* was detected *in vitro* assays. These results further confirmed the suitable biochar amendment was beneficial for the growth of microbes. But the biochar amendment at high concentrations suppressed the growth of *B. subtilis* also was detected, and this result may be caused by potential toxicity of excessive biochar. Previous studies have reported that biochar has potential toxicity for microbes with volatile organic chemicals and persistent free radicals (Fang et al., 2014; Zhu et al., 2017). While biochar amendment reduced the number of potential pathogen *Fusarium* spp. (Figure 6A), the distinct suppression of biochar on the *F. solani* was not detected in our *in vitro* assays. Such a result was similar to previous reported as biochar had not directly toxic effect on the pathogen, and can’t keep explaining that biochar amendment inhibited pathogens survival in the soil (Akhter et al., 2015; Jaiswal et al., 2014b, 2017). The number of gene copy is a sensitive parameter and can more clearly interpret the microbial responses to biochar amendment (Chen et al., 2013; Zhu et al., 2017). Based on real-time qPCR and the DNA copy numbers, our study detected that the pathogen *F. solani* survival in the natural soil was significantly decreased over increasing concentrations of biochar (Figure 8A), but there was not any the inhibitory effect of biochar amendment on *F. solani* in the sterilized soil (Figure 8C). Hence, we suggested that the inhibition of biochar amendment on pathogens survival in soil was an indirect process, which may be caused by stimulating beneficial microbes with antagonistic activity against pathogen, similar to previous suggestions (Elmer and Pignatello, 2011; Yang et al., 2019). Interestingly, the number of the *B. subtilis* survival in the natural soil and the sterilized soil were both increased over increasing biochar concentrations (Figures 8B,D). Here, it is suggested similarly as previous reports that biochar promoted the colonization of beneficial microbes *B. subtilis* at least partly was due to that biochar provide shelter with pore structures and surfaces for beneficial microbes to expand ecological niches (Quilliam et al., 2013; Kolton et al., 2016). In addition, based on previous reports (Joseph et al., 2013; Quilliam et al., 2013), biochar directly supplied nutrients with those nutrients on biochar particles and indirectly modified the properties of essential habitats for microbial growth also be considered as one of the major driving factors of biochar enriched the beneficial microbes. In short, biochar amendment can directly enrich beneficial microbe *B. subtilis*, but the suppression of biochar on the pathogen *F. solani* must be involved in beneficial microorganisms. As a result, we suggest that biochar application can alleviate negative plant-soil feedback is mostly due to that biochar amendment modified the soil microbiome, especially the suppression of pathogens caused by enriching beneficial microbes. Understanding the process of biochar application alleviate NPSF may help us better manage field crop cultivation for the purpose of sustainable soil management by modifying the soil microbiome to maintain soil health and mitigate the replanting problem in agricultural production. Moreover, previous studies have demonstrated that inoculation of microbial antagonists is an effective method that could control soil-borne diseases and alleviate NPSF (Dong et al., 2018; Luo et al., 2019). However, it is still an urgent problem in biological control of soil-borne diseases that to improve the ability of beneficial bacteria survive and colonize in soil. Hence, inoculation with beneficial microbe, such as *B. subtilis* with biochar as carrier can be considered as further study for the purpose of more effectively managing soil-borne disease and alleviating the replanting problem.

## Conclusion

We confirmed that biochar amendment can alleviate the NPSF by modifying soil microbiome. The results showed that biochar soil amendment altered the bacterial and fungal community compositions. Interestingly, we found that bacterial community was more sensitive to biochar amendment than fungal community. Biochar amendment significantly increased the bacterial community richness and diversity, but fungal community richness and diversity were not significantly affected by biochar. Moreover, we also found that biochar amendment improved soil chemical properties and those increased soil chemical variables positively correlate with the soil bacterial richness and diversity. In short, our study demonstrated that biochar application directly enriched beneficial microbe with the ability to inhibit pathogen and pathogen was indirectly suppressed in the soil, these eventually alleviated the NPSF. As a result, we suggested that biochar application should be considered as an environmental and efficient agricultural practice for sustainable soil management in agricultural ecosystem.

## Data Availability Statement

The datasets generated for this study can be found in the SRA database of NCBI (Accession Number: PRJNA578849), NCBI GenBank (Accession Number: MN520318), and NCBI GenBank (Accession Number: MN520103).

## Author Contributions

XH and YZ conceived the study and directed the project. WW, ZW, KY, PW, and HW performed the tests. LG and SZ gave critical comments and suggestions on an earlier version of this manuscript. XH and WW wrote the manuscript.

## Conflict of Interest

The authors declare that the research was conducted in the absence of any commercial or financial relationships that could be construed as a potential conflict of interest.

## Footnotes

1 www.majorbio.com
