# Supplementary material for: Biochar Application Alleviated Negative Plant-Soil Feedback by Modifying Soil Microbiome
Source: Front Microbiol. 2020 Apr 29;11:799. doi: 10.3389/fmicb.2020.00799 (PMC7201025; doi:10.3389/fmicb.2020.00799)
Supplement: Supplementary file 1 [file Table_1.DOCX]

Supplementary Material

# Supplementary Table

**Table S1** showed Illumina Miseq sequencing yielded 1071945 quality bacterial sequences with 112898-124375 bacterial sequences(mean=119105) per sample, after quality-filtering. B0, B0.5 and B2 stand for biochar soil amendment at a concentration of 0%, 0.5% and 2% (w/w), respectively.

**Table S1** Processed sample data information of 16s rRNA gene

| Sample ID | Number of Bases | Number of sequences | Mean length of sequences | Min length of sequences | Max length of sequences |
| --- | --- | --- | --- | --- | --- |
| B0-1 | 48441726 | 117543 | 412.1192 | 370 | 460 |
| B0-2 | 49888722 | 121203 | 411.6129 | 370 | 460 |
| B0-3 | 50270833 | 121951 | 412.2216 | 370 | 460 |
| B0.5-1 | 49333036 | 119585 | 412.5353 | 370 | 460 |
| B0.5-2 | 46526910 | 112898 | 412.1146 | 370 | 460 |
| B0.5-3 | 51276771 | 124375 | 412.2755 | 370 | 460 |
| B2-1 | 47473691 | 114703 | 413.8836 | 370 | 460 |
| B2-2 | 48337645 | 117260 | 412.2262 | 370 | 460 |
| B2-3 | 50605454 | 122427 | 413.3521 | 370 | 460 |
| average | 49128310 | 119105 | 412.4823 | 370 | 460 |
| total | 4.42E+08 | 1071945 | 3712.341 | 3330 | 4140 |
