# Supplementary material for: Biochar Application Alleviated Negative Plant-Soil Feedback by Modifying Soil Microbiome
Source: Front Microbiol. 2020 Apr 29;11:799. doi: 10.3389/fmicb.2020.00799 (PMC7201025; doi:10.3389/fmicb.2020.00799)
Supplement: Supplementary file 2 [file Table_2.DOCX]

Supplementary Material

# Supplementary Table

**Table S2** showed Illumina Miseq sequencing yielded 925965 quality fungal sequences with 101823-104726 fungal sequences per sample (mean=102885), after quality-filtering. B0, B0.5 and B2 stand for biochar soil amendment at a concentration of 0%, 0.5% and 2% (w/w), respectively.

**Table S2** Processed sample data information of ITS gene

| Sample ID | Number of Bases | Number of sequences | Mean length of sequences | Min length of sequences | Max length of sequences |
| --- | --- | --- | --- | --- | --- |
| B0-1 | 32160399 | 104726 | 307.0909 | 275 | 390 |
| B0-2 | 31598496 | 102419 | 308.5218 | 275 | 390 |
| B0-3 | 31479295 | 102002 | 308.6145 | 275 | 390 |
| B0.5-1 | 31744111 | 103169 | 307.6904 | 275 | 390 |
| B0.5-2 | 32627115 | 103587 | 314.9731 | 275 | 390 |
| B0.5-3 | 31115000 | 102389 | 303.8901 | 275 | 390 |
| B2-1 | 31766614 | 102439 | 310.1027 | 275 | 390 |
| B2-2 | 31597173 | 101823 | 310.3147 | 275 | 390 |
| B2-3 | 31627859 | 103411 | 305.8462 | 275 | 390 |
| average | 31746229 | 102885 | 308.5605 | 275 | 390 |
| total | 2.86E+08 | 925965 | 2777.044 | 2475 | 3510 |
