# Supplementary material for: Biochar Application Alleviated Negative Plant-Soil Feedback by Modifying Soil Microbiome
Source: Front Microbiol. 2020 Apr 29;11:799. doi: 10.3389/fmicb.2020.00799 (PMC7201025; doi:10.3389/fmicb.2020.00799)
Supplement: Supplementary file 4 [file Table_4.DOCX]

Supplementary Material

# Supplementary Table

**Table S4** showed the diversity indices and Good’s coverage of fungal community. B0, B0.5 and B2 stand for biochar soil amendment at a concentration of 0%, 0.5% and 2% (w/w), respectively. Data are shown as the mean with standard error. The means of alpha diversity indices were compared between treatments by the Tukey's honestly significant difference (HSD) test. Data in the same column followed by different letters showed significant differences among the treatments (p < 0.05; n = 3).

**Table S4** The diversity indices of soil fungi

| Treatment | Observed species | Shannon | Simpson | Chao1 | ACE | Goods coverage |
| --- | --- | --- | --- | --- | --- | --- |
| B0 | 282.00±10.58a | 3.71±0.26a | 0.80±0.03a | 290.55±12.74a | 294.04±12.76a | 1.000±0.000a |
| B0.5 | 253.00±3.06a | 3.25±0.30a | 0.74±0.08a | 277.61±6.12a | 278.55±6.07a | 1.000±0.000a |
| B2 | 260.33±2.03a | 3.32±0.14a | 0.72±0.02a | 273.89±5.88a | 276.08±5.13a | 1.000±0.000a |
