# Supplementary material for: Biochar Application Alleviated Negative Plant-Soil Feedback by Modifying Soil Microbiome
Source: Front Microbiol. 2020 Apr 29;11:799. doi: 10.3389/fmicb.2020.00799 (PMC7201025; doi:10.3389/fmicb.2020.00799)
Supplement: Supplementary file 5 [file Table_5.DOCX]

Supplementary Material

# Supplementary Table

**Table S5** The analysis of similarity (ANOSIM) based on Bray-Curtis distance at operational taxonomic unit level was performed using the free online platform of Majorbio Cloud Platform (www.majorbio.com). ANOSIM revealed significant differences in the structures of the bacterial and fungal community among different soils with biochar at the concentrations of 0%, 0.5% and 2% (w/w) (p < 0.05; n = 3), respectively. All permutation tests were conducted using 999 permutations.

**Table S5** the results of ANOSIM on bacterial and fungal community structures

| Analytic target | Method | Statistic (r) | P value | Permutation number |
| --- | --- | --- | --- | --- |
| Bacterial community | ANOSIM | 0.9342 | 0.001 | 999 |
| Fungal community | ANOSIM | 0.3333 | 0.017 | 999 |
