# Supplementary material for: Biochar Application Alleviated Negative Plant-Soil Feedback by Modifying Soil Microbiome
Source: Front Microbiol. 2020 Apr 29;11:799. doi: 10.3389/fmicb.2020.00799 (PMC7201025; doi:10.3389/fmicb.2020.00799)
Supplement: Supplementary file 6 [file Table_6.DOCX]

Supplementary Material

# Supplementary Table

**Table S6** showed the basic properties of biochar used in this study. The biochar, made from wheat straw at 500 ℃ pyrolysis temperature (Yunan Windsail Agricultural Tech CO., Ltd.), was used on the present research.

**Table S6** the basic properties of biochar used on the present research

| Basic Properties | Total Nitrogen (g/kg) | Available Nitrogen (mg/kg) | Available Phosphorus (mg/kg) | Available Potassium (mg/kg) | Organic Matter (g/kg) | Electrical Conductivity (μS/cm) | pH |
| --- | --- | --- | --- | --- | --- | --- | --- |
| Biochar | 1.59 | 95.55 | 349.34 | 5458.32 | 233.40 | 7463 | 9.47 |
